# Growth of *Cutibacterium acnes* is common on osteosynthesis material of the shoulder in patients without signs of infection

**DOI:** 10.1080/17453674.2018.1489095

**Published:** 2018-06-27

**Authors:** Anna Both, Till O Klatte, Andreas Lübke, Henning Büttner, Maximilian J Hartel, Lars G Grossterlinden, Holger Rohde

**Affiliations:** 1 Institut für Medizinische Mikrobiologie, Virologie und Hygiene;; 2 Klinik für Unfall- und Wiederherstellungschirurgie;; 3 Institut für Pathologie, Universitätsklinikum Hamburg-Eppendorf, Hamburg, Germany;; 4 Zentrum für Orthopädie, Unfall- und Wirbelsäulenchirurgie, Asklepios Klinik Altona, Hamburg, Germany

## Abstract

Background and purpose — *Cutibacterium acnes*, formerly known as *Propionibacterium acnes*, is often isolated from deep tissues of the shoulder. It is recognized as an important causative agent of foreign-material associated infections. However, the incidence and significance of its detection in tissues from patients without clinical evidence for infection is unclear. We assessed the incidence of *C. acnes* colonization of osteosynthesis material in asymptomatic patients, and evaluated the short-term outcome in relation to the microbiological findings.

Patients and methods — We microbiologically analyzed osteosynthesis material of 34 asymptomatic patients after surgery on the clavicle. Material obtained from 19 asymptomatic patients after osteosynthesis of the fibula served as a control group. Patients were clinically followed up for 3–24 months after removal of the osteosynthesis material.

Results — Bacteria were recovered from devices in 29 of 34 patients from the clavicle group. 27 of 29 positive samples grew *C. acnes*. Isolation of *C. acnes* was more common in male than in female patients. No bacterial growth was observed on foreign material from patients in the fibula group. All patients remained asymptomatic at follow-up.

Interpretation — Growth of *C. acnes* is common on osteosynthesis material of the shoulder, especially in males. Samples were positive irrespective of clinical signs of infection. Therefore, detection of *C. acnes* in this clinical setting is of questionable clinical significance. The high positivity rate in asymptomatic patients discourages routine sampling of material in cases without clinical evidence for infection.

Operative treatment of shoulder fractures has increased since the early 2000s (Persico et al. 2014). Infections after operative fracture-fixation occur with an incidence of roughly 3%, although large prospective studies are lacking. Similarly, prosthetic joint infections (PJI) of the shoulder occur with an incidence of 3%, comparable to PJI of the hip and knee (Kurtz et al. 2012, Bohsali et al. 2017).

In recent years, most likely due to improved diagnostic procedures, an increase in *Cutibacterium acnes* infections has been noted (Achermann et al. 2014, Shifflett et al. 2016). *Cutibacterium acnes*, formerly *Propionibacterium acnes*, is a Gram-positive anaerobic rod-shaped bacterium, commensally inhabiting the pilosebaceous unit in humans. Though a part of the normal human skin microbiota, *C. acnes* is also implicated in biofilm-associated infections and inflammatory processes, such as prosthetic valve endocarditis, infection of breast or eye implants, and acne vulgaris (Aubin et al. 2014, Beylot et al. 2014, van Valen et al. 2016). Of note, *C. acnes* is increasingly recognized as an important pathogen in bone and joint infections (Achermann et al. 2014). The shoulder is the most commonly affected joint in *C. acnes* infections. In fact, *C. acnes* has previously been isolated in one-quarter of revision arthroscopies for shoulder pain or stiffness after a first arthroscopy (Horneff et al. 2015). Even more strikingly, *C. acnes* has been identified as the most frequently isolated pathogen in prosthetic shoulder joint infections (Piper et al. 2009, Kadler et al. 2015).

*C. acnes* can lead to infections that are clinically evident, while some cases with detection of *C. acnes* are considered only mildly symptomatic or even asymptomatic. One study found 42% of joint fluid aspirates taken at primary shoulder replacement to be positive for *C. acnes*, and in another study 20% of deep tissue specimens acquired after shoulder arthroscopy grew *C. acnes,* both studies focusing on patients with no clinical signs of infection (Levy et al. 2013, Chuang et al. 2015). Consequentially, there has been a debate over the implication of growth of *C. acnes* in tissue samples from shoulders in the absence of clinical signs of infection.

In this study we assessed the frequency of bacterial colonization of osteosynthesis material in healthy adults with no signs of foreign material infection and assessed self-reported outcomes at 3 to 24 months of patients with osteosynthesis material, which were culture positive, compared with those who had sterile devices.

## Patients and methods

### Patient selection and sample collection

Inclusion criteria were age over 18 years with need for removal of a fixation device of the clavicle or distal fibula without any clinical signs of infection. Except for the hook plate, which has to be removed routinely, the reason for removal of the osteosynthesis material was at the patient’s request, mainly due to foreign body sensation or for cosmetic reasons.

Patients in the clavicle group (n = 34) had suffered a closed clavicle fracture or a closed dislocation of the acromioclavicular (AC) joint due to trauma. In case of a lateral clavicle fracture (n = 7) or dislocation of the AC joint (n = 12), open reduction and fixation with a hook plate was performed. To avoid any complication due to the design of this plate routine removal was considered necessary after 4 months in the case of AC joint dislocation and after 6 months in the case of a lateral clavicle fracture. The other clavicle fractures (n = 15) were treated by open reduction and internal fixation with a standard plate.

All patients in the fibula group (n = 19) had a closed fracture of the distal fibula, which was treated with open reduction and internal fixation by plate and screws.

Exclusion criteria were development of pseudarthrosis, intake of antibiotics 2 weeks before removal of the fixation device for any reasons, presentation of fistula or abscess in the area of the fixation device, an open injury of the clavicle or fibula, any history of wound healing disturbance, or infection of the fixation device after the initial surgery.

Preoperatively physical examination was undertaken and C-reactive protein levels (CRP) in serum and leucocyte count were assessed in every patient.

Removal of the osteosynthesis material was performed in the clavicle and fibula group similarly. The surgical region was shaved where appropriate. For skin disinfection isopropanol was applied for 8 minutes. The skin was allowed to dry; thus isopropanol had a total exposure time of 10 minutes. After skin incision, the surgical blade was changed and the fixation device dissected. Two tissue specimens of around 0.5 x 0.5 x 0.5 cm were taken above and 1 below the fixation device. Plates and screws were separately transferred to sterile containers and immediately transported to the microbiological laboratory for testing. Perioperative antibiotic prophylaxis in the form of 2 g intravenous cefazolin was given after all samples were taken.

Patients were seen postoperatively once at the first postoperative day for routine check-up. Finally, 3 to 24 months after surgery, participating patients were called by telephone and asked if any further pain, limitation of motion, foreign body sensation, or problems with wound healing had occurred.

### Microbiology

Tissue samples from under and above the fixation plate were transferred to a sterile mortar and covered with 1 mL of sterile phosphate-buffered saline (PBS). Tissues were homogenized and 100 µL of the suspension was plated on Columbia sheep blood agar, chocolate agar, Sabouraud agar, and Schaedler anaerobic agar (all Oxoid, Basingstoke, UK). Plates were incubated at 36 °C in 5% CO_2_ or under anaerobic conditions. Plates were read after 48 h, 7 days, and 14 days. Additionally, 2 mL of thioglycolate broth was inoculated with one drop of the tissue suspension and incubated likewise.

Osteosynthesis screws were transferred to a sterile 50 mL Falcon tube (Greiner, Frickenhausen, Germany) and plates were transferred to a sterilized implant box (Lock & Lock, Seoul, South Korea) in a biosafety cabinet with laminar airflow. Screws were covered with 10 mL and plates were covered with 50 mL of PBS, respectively. These air-tight sealed containers were vortexed at maximum speed for 30 s. Vessels containing the foreign material were treated in an ultrasound bath for 3 min at 40 kHz (BactoSonic; Bandelin GmbH, Berlin, Germany) followed by 30 s of vigorous shaking.

Sonicate fluid was transferred into new Falcon tubes and centrifuged at 3500 rpm for 10 min. 9 mL and 49 mL of supernatant, respectively, was removed and the pellet was resuspended by pipetting in the remaining 1 mL of fluid. Sheep blood agar, chocolate agar, Sabouraud agar, and Schaedler anaerobic agar were each inoculated with 100 µL of the sonicate fluid. Plates were incubated and read as described above.

For histological analysis, 11 tissue samples from below the fixation device from 9 clavicular group patients and 3 fibula group patients were placed into 4% formalin and embedded into paraffin blocks.

### Statistics

Categorical variables, such as surgical site, sex, restriction of movement, and foreign body sensation were tested for their association with having positive cultures from tissue or foreign material by Fisher’s exact test.

### Ethics, funding, and potential conflicts of interest

Internal review board approval was obtained (No: PV4696, Review Board of University Medical Center Hamburg-Eppendorf). All participants gave written informed consent. The study was supported by the Damp Foundation (project 2013-19). There are no conflicts of interest to declare.

Overview of patients with growth of non-*C. acnes* bacterial species

**Table:** 

Patient	Growth on foreign material	Growth in peri-implant tissue	Interpretation
3	*C. acnes*	*C. acnes*	
	*S. saccharolyticus* (low CFU **^a^**)	*S. saccharolyticus* (low CFU ^a^)	
6	*C. acnes*	*C. acnes*	Contamination likely**^b^** (*K. rhizophila; Brevundimonas sp.*)
	*K. rhizophila* (low CFU **^a^**)		
	*Brevundimonas sp.* (low CFU **^a^**)		
9	*C. acnes*	*C. acnes*	
	*S. epidermidis*	*S. epidermidis*	
30	*C. acnes*	*C. acnes*	Contamination likely**^b^** (*P. mirabilis; S. capitis, S. epidermidis*)
	*P. mirabilis* (low CFU **^a^**)	*P. mirabilis* (low CFU)	
	*S. capitis* (low CFU **^a^**)		
	*S. epidermidis* (low CFU **^a^**)		
36	*C. acnes*	*C. acnes*	Contamination likely**^b^** (*S. saccharolyticus*)
	*S. saccharolyticus* (low CFU **^a^**)		
18	*S. epidermidis* (low CFU **^a^**)	No growth	Contamination likely**^b^**
45	*S. saccharolyticus*	*S. saccharolyticus*	

**^a^**Low CFU indicates colony count of under 10 CFU/mL in sonicate fluid.

**^b^**Contamination was deemed likely if species were detected in only one of the analyzed materials (fixating plate, screws, tissues from above

or below the fixation devices).

## Results

### Patient characteristics

53 patients (median age 41 years [21–74], 36 men) undergoing removal of osteosynthesis material between February 2016 and September 2017 were included in the study. 34 patients underwent removal of osteosynthesis material from the clavicle and 19 patients had material removed from the fibula. The foreign material was removed after a median of 6 months (3–120) for clavicular osteosynthesis and 20 months (8–53) for fibular osteosynthesis. Median leucocyte count in blood samples at a mean of 14 days (1–67) before surgery was 6.8 Giga/L (3.7–12). Median C-reactive protein levels were <5 mg/L (< 5–50).

### Isolated organisms

Tissue samples, as well as screws and fixating plate, were available for microbiological analysis in all patients. Bacterial growth was observed in 29/34 of tissue samples from clavicular osteosynthesis. 27 samples from the clavicle group grew *C. acnes*. Interestingly, only 1 out of 4 female patients undergoing removal of a fixation device from the clavicle had cultures positive for *C. acnes*, while 26 out of 30 male patients had cultures positive for *C. acnes* (p = 0.02). Cultures from sonication fluid of clavicular foreign material gave largely similar results to the tissue culture for *C. acnes*. In 1 patient *C. acnes* was found only on the foreign material but not in the surrounding tissue.

22 of those samples grew only *C. acnes*, while 5 showed growth of at least one additional organism (Table). There were no sex-specific differences in the growth of non-*C. acnes* isolates. In Patient 3 *C. acnes* was accompanied by *S. saccharolyticus*, and materials from Patient 9 showed growth of *S. epidermidis* in addition to *C. acnes*. In both patients 3 and 9 the respective second species was present in multiple materials and high colony counts.

Patient 30 showed additional growth of *S. epidermidis* and *S. capitis* on the osteosynthesis material and *P. mirabilis* in 1 tissue sample, while samples from Patient 6 showed additional growth of *Brevundimonas sp*. and *Kocuria sp*. Foreign materials of Patient 36 grew *S. saccharolyticus* in addition to *C. acnes*. The non-*C. acnes* species in Patients 6, 30, and 36 were grown in very low numbers, thus they might constitute contamination during removal from the body or microbiological workup (Table).

Samples from Patient 45 grew only *S. saccharolyticus*. Osteosynthesis material from Patient 18 grew *S. epidermidis* in very low numbers, which might also, rather, constitute contamination.

Neither tissue samples nor osteosynthesis materials from any of the 19 patients who underwent removal of fibular osteosynthesis showed bacterial growth.

Patients whose specimens grew *C. acnes* were not more likely to have a leucocyte count or serum CRP above the median than patients with sterile specimens (p = 1.0).

Tissue samples from below the fixation device from 8 clavicular group patients and 3 fibula group patients were analyzed in histology. All samples showed signs of fibrosis and all but 1 showed small to moderate amounts of metal particles. Two samples from clavicular group patients showed few to moderate signs of inflammation. Microscopy of tissue from Patient 51 revealed 25 polymorphonuclear leucocytes (PMN) per 10 high power fields (HPF). No systemic signs of infection were noted (blood leucocyte count: 3.7 Giga/L, CRP <5 mg/L). There was no growth of aerobic or anaerobic bacteria in either tissue or on the foreign material. Histology of tissue from Patient 36 showed 55 PMN per 10 HPF. Blood leucocytes were normal at 6.4 Giga/L; CRP was <5 mg/L. Cultures from foreign material and tissue grew *C. acnes*, additionally small amounts of *S. saccharolyticus* were cultured from the foreign material. Of the remaining 6 samples from the clavicular group, without signs of inflammation, 3 had positive cultures for *C. acnes* and 3 were culture negative. None of the 3 samples from the fibula group showed signs of inflammation.

During the follow-up telephone call at 3 to 24 months postoperatively, none of the patients complained about pain, limitation of motion, foreign body sensation, or problems with wound healing.

## Discussion

We found a high incidence of bacterial growth on osteosynthesis material of the clavicle in patients with no clinical signs of infection. In a control group of patients undergoing removal of osteosynthesis material devices of the fibula, no bacterial growth was observed. *C. acnes* was by far the most common organism isolated from the foreign material. Of note, materials from male patients were significantly more likely to have positive cultures for *C. acnes* compared with specimens from female patients. This finding is limited by the small number of women included in this study, which is due to the epidemiology of shoulder injuries (Kihlström et al. 2017). In support of our findings, however, others have found a higher incidence of *C. acnes* infection in men as compared with women (Berthelot et al. 2006, Millett et al. 2011). A possible explanation for this finding is that men are more commonly colonized with *C. acnes* in the pilosebaceous units of the head, neck, shoulders, and upper trunk (Kadler et al. 2015).

Prosthetic shoulder joints are at special risk for infection with *C. acnes*, which is much less common in knee or hip prosthetic joint infections. In fact, *C. acnes* was found to be the most commonly or second most commonly isolated organism in shoulder prosthetic joint infections in many studies or case series examining revision shoulder arthroplasties (Pottinger et al. 2012, Nelson et al. 2016, Singh et al. 2012, Wang et al. 2013). Interestingly, *C. acnes* has been implicated in so-called aseptic loosening of prosthetic joints and glenohumeral arthropathy, both without any overt signs of infection (Levy et al. 2013). It may be that *C. acnes* preferentially establishes slowly destructive infections that do not necessarily elicit an inflammatory response.

However, the interpretation of the pathogenic significance of *C. acnes* in tissue samples especially from the shoulder is challenging. Some reports suggest an elevated risk of contamination during incision or removal of the specimen in this location (Hudek et al. 2014), potentially promoted by the sometimes insufficient removal of *C. acnes* from the dermis by surgical skin preparation (Lee et al. 2014). Building on the frequent identification of *C. acnes* in apparently non-infected joints, others even speculated that the organism might be able to colonize the joint without clinical signs of infection (Zeller et al. 2007, Chuang et al. 2015). In line with these observations made in shoulder arthroplasty, in our series of patients a high proportion of fixation devices of the clavicle were positive for *C. acnes*, but clinical signs of infection and histologic evidence suggestive of infection were lacking. Moreover, no significant difference in laboratory infection parameters in patients positive with *C. acnes* on the foreign material and those with negative specimens were found. Thus, the idea is supported that, due to its innocuous nature, *C. acnes* indeed can colonize even body sites and tissues that usually would be regarded as sterile, and may persist even for long periods without causing signs of infection. However, clinical significance is extremely difficult to assess as even manifest infections with *C. acnes* may show only slight signs of infection, i.e., systemic parameters such as leucocyte count, erythrocyte sedimentation rate, and C-reactive protein in serum may be normal (Uçkay et al. 2010, Piggott et al. 2016). Importantly, in our study, no significant histologic signs of infection were observed, and all patients were well at follow-up. Also important is that the presence of an implant has been found essential for persistent *C. acnes* infections in an animal model of foreign-body infection (Shiono et al. 2016). Therefore, it cannot be ruled out that *C. acnes* positive patients would have developed apparent infections at later time points if devices were not removed. However, in our experience and after reviewing the literature, complications of osteosynthesis after more than one year appear to be rare.

In summary, detection of *C. acnes* in tissue samples from osteosynthesis surgery of the clavicle must be interpreted with caution, even if high numbers of bacteria are recovered. Microbiological sampling should always be accompanied by histopathological analysis, and possibly additional markers like α-defensin or C-reactive protein, allowing for complete assessment of possible infection. As long as no specific markers to differentiate between invasive and contaminating isolates are available, it is advisable not to send tissue for microbiological analysis if no clinical signs or symptoms suggestive of infection are present. Our data suggest that, otherwise, unnecessary antibiotic treatment of uninfected individuals could occur. Further studies addressing the role of *C. acnes* in surgery-related complications like pseudarthrosis are warranted.

AB, TOK, HR, and LGG designed the study, AB, TOK, HR, AL, HB, and MJH conducted the experiments and collected and interpreted the data. HR and AB wrote the manuscript.

The authors would like to thank Claudia Lehnert for the excellent technical support.

*Acta* thanks Lars Gunnar Johnsen and other anonymous reviewers for help with peer review of this study.
